# Effectiveness of Parameters in Quantifying Root Canal Morphology Change after Instrumentation with the Aid of a Microcomputed Tomography

**DOI:** 10.1155/2019/9758176

**Published:** 2019-07-02

**Authors:** Alexander Juhasz, Csaba Hegedus, Ildiko Marton, Balazs Benyo, Kaan Orhan, Csaba Dobó-Nagy

**Affiliations:** ^1^Department of Restorative Dentistry, Faculty of Dentistry, University of Debrecen Medical and Health Science Center, Debrecen, Hungary; ^2^Department of Biomaterials and Prosthetic Dentistry, Faculty of Dentistry, University of Debrecen Medical and Health Science Center, Debrecen, Hungary; ^3^Department of Control Engineering and Information Technology, Budapest University of Technology and Economics, Budapest, Hungary; ^4^Ankara University, Faculty of Dentistry, Department of Dentomaxillofacial Radiology, Ankara, Turkey; ^5^OMFS IMPATH Research Group, Department of Imaging & Pathology, Faculty of Medicine, University of Leuven and Oral & Maxillofacial Surgery, University Hospitals Leuven, Leuven, Belgium; ^6^Department of Oral Diagnostics, Department of Oral Diagnostics, Faculty of Dentistry, Semmelweis University, Budapest, Hungary

## Abstract

The objective of this study was to analyse the effectiveness of some parameters which characterise the change in morphology in human root canals subjected to ProTaper rotary enlargement with the help of an X-ray microfocus computed tomography (MCT) and to introduce a novel parameter that is effective in quantifying changes in root canal morphology. Ten each straight and curved root canals with mature apices chosen from extracted human upper incisor and canine teeth were scanned with MCT before and after canal shaping using ProTaper rotary instruments in order to facilitate three-dimensional digital reconstruction and quantitative gauging of relevant instrumental parameters and changes therein (surface area and volume). Root canal geometry change and the effectiveness of shaping were quantified with Structure Model Index change (ΔSMI) and surface area change to volume change ratio (ΔSA/ΔV). These two parameters were also tested on simulated canals. Postinstrumentation cross-sectional changes were also analysed, but only on the plastic blocks. Statistical analysis of parameters was carried out to verify the significance of results. Analysis of cross-sectional shape of postinstrumented resin simulated canals showed statistically significant decrease in Form Factor (p<0.05) and statistically significant increase in Eccentricity (p<0.005). ΔSMI did not show significant difference between straight and curved canals. SMI values showed bidirectional change during root enlargement which questions the reliability of this metric in analysing instrumentation. Statistically significant (p<0.005) deviations in ΔSA/ΔV were quantified as 1.92 and 3.22 for straight and curved human canals, respectively. Instrumentation-induced canal geometry change was determined to be more pronounced in curved canals using the novel parameter ΔSA/ΔV. This has been proven as being a statistically accurate and reproducible parameter for quantitative characterisation of root canal geometry change and differentiation of preparational efficacy for both straight and curved root canals.

## 1. Introduction

Root canal instrumentation is strongly affected by canal configuration [1] with studies showing morphology to be highly influential on the efficacy of canal preparation. Frequency and magnitude of canal aberrations (e.g., zip, elbow, perforations, and asymmetric preparation) have been proven as being more prominent in curved root canals than straight ones [2].

X-ray microfocus computed tomography (MCT) has been extensively applied as a reliable methodology for the quantitative evaluation of root canal instrumentation [3, 4]. And for the evaluations the following parameters have been used to characterise the quality of instrumentation previously: surface area change (ΔSA), volume change (ΔV), Structure Model Index change (ΔSMI) and Centre of Mass change (CM shift). The ΔSMI parameter reflects the cross-sectional change in the root canal after instrumentation, especially for oval and round cross-sectional root canals. The natural root canal has concave and convex surface irregularities. Nevertheless, a simplified equation for the SMI for root canals has been proposed as

(1) $$\begin{array}{c} SMI=\frac{6S^{\prime }.V}{S^{2}} \end{array}$$

where S is the root surface area preinstrumentation; S' is the change in the surface area caused by instrumentation; and V is the original volume of the root canal preinstrumentation [5].

Surface dilatation smooths the surface irregularities and this will reduce surface area S in equation (1) causing S' to be negative. For root canals one can expect that the SMI value change after instrumentation reflects both oval to round cross-sectional transformation and the smoothing of the root canal walls.

The root surface area (SA) and root volume (V) have been proven as being demonstrative of the canal shape, both pre- and postinstrumentation [3, 4, 6, 7]. However, when compared with experimental groups, contradictory results have been found in the changes of these parameters, ΔSA and ΔV. In a MCT study [8] reported no significant differences regarding 3D parameters of root canal preparation while on the same teeth most of 2D parameters delivered statistically significant differences between groups. These results raise a question on usefulness of three-dimensional parameters describing canal changes during enlargement.

Therefore, the objective of this study was to quantitatively evaluate the change of root canal morphology after instrumentation with the ratio of surface area change to volume change ΔSA/ΔV, in comparison with SMI, with the aid of MCT.

## 2. Materials and Methods

### 2.1. Sample Selection and Pretreatment

Ten each straight and curved root canals with mature apices were selected from forty-three extracted human upper incisors and canine teeth due to severe bone loss for the study. Schneider's classification [9] was used as the grouping criterion, where root canals with <10° and >25° curvature were designated as straight and curved, respectively. Teeth with mature apices having one main root canal without obliteration were included. Teeth having between 11° and 24° curvature were excluded. Pulp remnants were removed from each root canal using barbed broaches without any canal preparation. The teeth were then stored in 0.1% thymol solution.

### 2.2. Root Canal Preparation of Natural Human Teeth

All of the twenty chosen root canals were prepared using the ProTaper protocol with a torque-controlled, constant speed endomotor (Technika, Dentsply-Maillefer, Switzerland). The SX shaping file was used first to provide an initial flare to the orifices. An ISO 10 K-reamer (Dentsply-Maillefer, Switzerland) was used with light downwards “watch winding” motion to determine the overall working length, which was calculated by subtracting 0.5 mm from the length reading when the tip was just visible at the main apical foramina of a root. ProTaper rotary files (both shaping and finishing) were then used in “pecking” motion in the following sequences: S1 and S2 were advanced to resistance but no more than two-thirds of the canal depth; SX was then introduced into the canal in “brushing” motion to 3-5 mm short of the working length followed by the use of S1 and S2 at the working length; and finally F1 and F2 finishing files were used at the working length.

Histolith 2.5% NaOCl solution (Lege Artis, Germany) was used for irrigation with a 31-gauge needle after each file use and 30 ml in total was used for each root canal. Glyde (Dentsply-Maillefer, Switzerland) was used as lubricant, with the amount enough to cover all the flute area of each file. Canal recapitulation was also performed after the use of each file. Files were regularly wiped using wet gauze to remove debris. A final flush was carried out using 5 ml sterile saline solution before drying with F2 ProTaper paper points (Dentsply-Maillefer, Switzerland). All instrumentation was performed according to the manufacturer's instructions. In order to reduce interoperational variability all preparation was conducted by the same operator. In order to reduce contamination, one set of instruments was only used for the preparation of one canal. The study was approved by the Regional and Institutional Committee of Science and Research Ethics of Semmelweis University, Budapest, Hungary (IRB ID: 246/2017).

### 2.3. Preparation of Simulated Canals

Twenty transparent resin simulated root canal blocks were used to assess the ProTaper instrumentation, amongst which ten were straight (A-ETK-0 endotrainer, Frasaco, Tettnang, Germany) and ten were curved with a curvature of 30° (A-ETK-30 endotrainer, Frasaco, Tettnang, Germany).

Root canal preparation was carried out in the same way as was described for natural human teeth except that Glycerine was used as lubricant instead of Glyde.

### 2.4. MCT Scanning of the Samples

All samples, both natural human teeth and resin simulated root canal blocks, were scanned before and after instrumentation with a desktop X-ray microfocus computed tomography scanner (SkyScan 1172, Bruker, Kontich, Belgium) at 10 W, 100 kV, and 98 *μ*A, with a 0.5 mm aluminium filter, resulting in an isometric voxel size of 9 *μ*m. During data acquisition, 2D projections through 180° of rotation were stored in digital format on an electronic media. The 3D images were obtained by filtered back-projection of a series of 2D images of adjacent cross-sections.

### 2.5. Data Analysis

#### 2.5.1. Volumetric Analysis

CTAn software (Bruker, Kontich, Belgium) was used to measure surface area (SA) and volume (V) after manual threshold segmentation of the root canal systems. Surface area change (ΔSA) and volume change (ΔV) were then calculated by subtracting the measured values of the untreated canals from those of the treated ones. The ratio of the surface area change to volume change ΔSA/ΔV was then calculated accordingly. Triangulated data were also used to determine the Structure Model Index (SMI) of the canals. This index characterises the cross-sectional geometry of the root canal as having a plate-like shape when SMI = 0, and a rod-like shape when SMI > 3 [10]. For statistical analysis of results Mann-Whitney U test in case of natural human roots and in case of plastic blocks Kruskal-Wallis and Tukey-HSD post hoc tests were used. Power analysis was done where the case of prepared sample size was 12 (six each group); the value of the power was 0.744. Based on this calculation, it can be ascertained that, in a sample size of 20, the value of the power exceeds 0.8.

#### 2.5.2. Cross-Sectional Analysis

In order to track the eccentric movements of the files, the cross-sectional shape at 14 mm coronal from the apex of the straight resin simulated canals was analysed with the CTAn software (Bruker, Kontich, Belgium). Form Factor, Roundness, and Eccentricity values were used to describe the canal cross-sectional shape changes caused by canal instrumentation. Statistical analysis was performed with one-way ANOVA and two-sample t-test.

## 3. Results and Discussion

**Figure 1:**
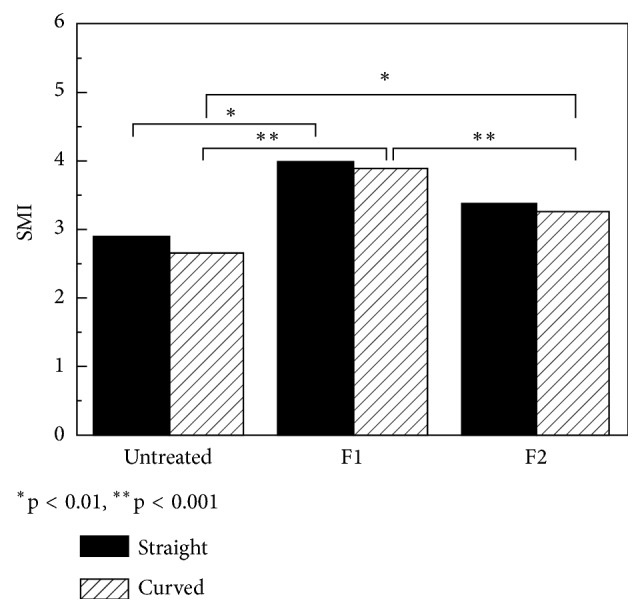
Bidirectional movement of SMI values, demonstrated by instrumentation on straight and curved resin simulated root canals. Bars represent statistical differences at *∗*p<0.01 level and *∗∗*p<0.001 level amongst groups of untreated, F1 prepared, and F2 prepared roots.

The present study focused on the quantitative characterisation of the difference in canal morphology using available parameters. Despite previous studies [4, 6, 10] showing the usefulness of SMI tracking, it did not prove effective in the current work. Our observation is in line with some recently published results [11, 12] where significant differences in SMI between tested groups could not been observed. The mean values (standard deviation) of the ratio of surface area change to volume change and SMI change after the ProTaper instrumentation in straight and curved natural canals are tabulated in Table 1. It is seen that ΔSA/ΔV values are not statistically different amongst the straight canals but are statistically different amongst the curved canals (p<0.05) and are even more statistically different between the two groups (p=0.01) (power=1.00). On the contrary, ΔSMI values are not significantly different within each group nor between the groups (p=0.74) (power=0.89). Unlike ΔSA/ΔV, the SMI did not change significantly after canal instrumentation in straight nor curved human canals (Table 1). The limitation of SMI in describing root canal morphology change after instrumentation is inherent in the bidirectional change of SMI value (Figure 1). Figure 1 demonstrates an increasing movement first at F1 files and then a decreasing movement at F2 files of SMI values during instrumentation. Specifically, the cross-sectional shape change from round to irregular after eccentric instrumentation reduces the SMI value, while smoothing the canal walls increases the SMI value. These opposite processes cause fluctuation in SMI values, resulting in the loss of significance in statistics.

**Figure 2:**
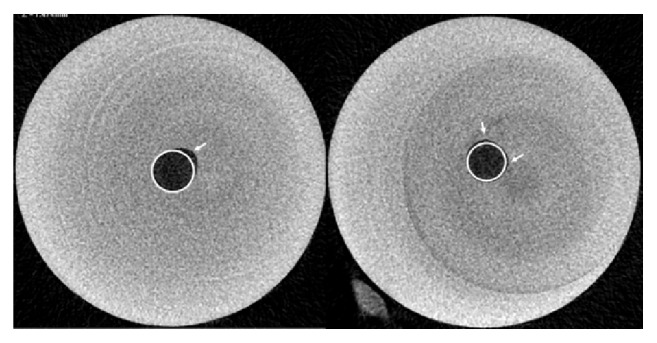
Cross-sectional images of prepared straight canals at 14 mm coronal from the apex in plastic blocks. White circles represent the regular round shape and white arrows point at contours of the canals showing different levels of irregularity.

Our experiments carried out on resin blocks helped to reduce the influence of the bidirectional change on SMI because preparation of artificial canals excluded the effect of canal wall smoothing since the canal walls prior to preparation were already completely smooth. So the only effect was the round to near-oval cross-sectional shape change due to eccentric movement of the files. This finding was in line with a previous observation [13]. The mean values (standard deviation) of the ratio of surface area change to volume change and SMI change after the ProTaper instrumentation in straight and curved resin simulated root canals are tabulated in Table 2. ΔSA/ΔV values are statistically different both within and between the two groups (p<0.001). ΔSMI values are statistically different within the curved group (p<0.01) but are not significantly different within the straight groups nor between the groups. Therefore, SMI value changes (Table 2) of resin simulated root canals reflect solely the deformity of cross-sectional shape, while those of natural teeth reflect both the cross-sectional deformity of the canals and the smoothing of the canal walls. Cross-sectional shape change of straight resin root canals showed that even in these simplified morphology the operator usually prepares one side of the root canal wall more effectively than the others. Consequently, the cross-sectional shape of prepared root canals changed from regular to irregular, resulting in Eccentricity (Figure 2). This finding was also reflected in the trend of the determined Form Factor and Eccentricity values (Table 3). When pre- and postinstrumentation values are compared, it is seen that after the instrumentation the Form Factor decreased significantly (p<0.05), the Eccentricity increased significantly (p<0.005), and the Roundness value did not show statistical difference.

Limitations of 3D analysis of root canal shape changes are inherent in bidirectional changes of SMI values during the enlargement of root canals as explained above. And on the other side the sample size of natural human roots may have an importance since the ideal size of sample is suggested to determine at a lower sample size of during the experiment. In this study at n=6 the power analysis resulted in 0.74 of which calculation predicted n=10 as an acceptable final sample size. It can be interesting note that another research group used 11 and 40 sample sizes (almost four times magnitude difference) in their different publications when using the same 3D analysis on natural root canals [4, 6].

Similar to bone trabecular, the natural root canals have concave and convex surface irregularities. Thus the SMI value increases after instrumentation if the original root canal walls are smooth and decreases if they are rough. The summation of this bidirectional change of SMI value is hardly predictable and this is why SMI value change due to root canal preparation is statistically different in some canals but not in others [8, 14]. In our study on natural human root canals statistically significant differences were not found in ΔSMI but in ΔSA/ΔV (Table 1). We have demonstrated the bidirectional change of SMI, for the first time ever to the best of our knowledge, on resin blocks where an initial increase of this parameter was followed by a decrease (Figure 1).

Structure Model Index (SMI) [5] was proposed to provide a quantified measure of the architectural type of cancellous bone in 1997. Since then SMI has become a widely used parameter to characterise the rod- and plate-like structure of 3D trabecular bone images. Naturally, SMI calculation is based on dilatation of the analysed bone structure, giving the surface area change relative to the dilatation (Δr), i.e., the surface area derivative (dS(r)/dr) by the dilatation distance (Δr). Accordingly, the SMI is calculated from

(2) SMI=6·VS2·dSrdr

(3) SMI=limΔr→0⁡6·VS2·ΔSrΔr

where S is the surface area of the analysed structure, ΔS(r) is the surface area change as a result of dilatation by Δr, and V is the bone volume.

In practical applications a triangular mesh is normally fitted to the image of the bone surface [6] so the bone surface area is approximated as the sum of the triangular mesh. Then the mesh is expanded by a short distance (Δr) away from the surface in the direction of the mesh's vertex normals. Δ*S*(r) is calculated as the difference between the expanded surface and the original surface. Some applications like CTAn software [15] use a voxelized representation of the bone structure and the dilatation is implemented by adding an additional voxel layer to the surface of the volume using the classical morphological image processing step of dilatation. However, the calculated SMI with the alternative implementations are closely correlated [16].

Since, besides the proportions of the rod- and plate-like regions, the SMI is also strongly influenced by the proportions of the concave and convex surfaces, the calculated SMI may show misleading value when the proportion of the concave surface is not negligible relative to the entire bone surface. In the cases where the bone structure is modelled by rods- and plates-like elements, concave surfaces are likely to be generated in the places where these elements join. Due to this increase in the proportion of concave surface the SMI may fail to characterise trabecular architecture of the bone, as has been demonstrated by Salmon et al. [16]

Beside the areas where rod- and plate-like elements are joining with each other, concave surfaces can also be found in the bone structure if the resolution of the imaging modality used to study the bone is high enough to capture the surface irregularities. Consequently, the higher resolution of the applied imaging modality the higher possibility there will be for SMI to show misleading trend. This limitation of the SMI index has already been recognised by Hildebrand and Rüegsegger where the derived values corrupted when a bone structure with a rough surface was analysed [5]. In order to eliminate this limitation, these authors then suggested smoothing the surfaces prior to the calculation of volume, surface, and surface derivative by locally averaging the vertices.

Hildebrand and Rüegsegger also predicted that SMI analysis using high resolution may encounter the same problem [5]. Since the microCT modality provides high resolution image sets of root canals with uneven surfaces could be inherent in this problem. The current work proved the prediction of Hildebrand and Rüegsegger to be true with experimental results and showed that the limitation of SMI also existed when applied to the analysis of root canal morphology.

## Figures and Tables

**Table 1:** The mean values (standard deviation) of the ratio of surface area change to volume change and SMI change after the ProTaper instrumentation in straight and curved natural canals.

Groups	ΔSA/ΔV	ΔSMI
Straight	*2.05 * ** **ns (0.88)	*0.65 * ** **ns (1.00)
Curved	*3.23∗* (0.30)	*0.44 * ** **ns (0.47)

Significance between groups	*∗∗*	ns

^*∗*^Statistically significant at p<0.05 level.

^*∗∗*^Statistically significant at p≤0.01 level.

ns: not statistically significant (n=10).

**Table 2:** The mean values (standard deviation) of the ratio of surface area change to volume change and SMI change after the ProTaper instrumentation in straight and curved resin simulated root canals.

Groups	ΔSA/ΔV	ΔSMI
Straight	*6.13∗∗* (1.11)	*0.86 * ** **ns (0.63)
Curved	*7.28∗∗* (0.79)	*0.74∗* (0.39)

Significance between groups	**∗** **∗**	ns

^*∗*^Statistically significant at p<0.01 level.

^*∗∗*^Statistically significant at p<0.001 level.

ns: not statistically significant (n=10).

**Table 3:** Cross-sectional shape of straight resin simulated root canals at 14 mm coronal from the apex: mean values (standard deviation) of Form Factor, Roundness, and Eccentricity pre- and postinstrumentation with the ProTaper system.

Groups	Form Factor	Roundness	Eccentricity
Preinstrumentation	*0.898* (0.039)	*0.937* (0.047)	*0.08* (0.091)
Postinstrumentation	*0.833* (0.027)	*0.901* (0.04)	*0.24* (0.044)

Significance between Groups	**∗**	ns	**∗** **∗**

^*∗*^Statistically significant at p<0.05 level.

^*∗∗*^Statistically significant at p<0.005 level.

ns: not statistically significant (n=10).

## Data Availability

The [xls files] data used to support the findings of this study were supplied by the corresponding author under license and so cannot be made freely available. Request for access to these data should be made to Csaba Dobo-Nagy, dobonagy.csaba@dent.semmelweis-univ.hu.
